# Fine-tuning AMPK in physiology and disease using point-mutant mouse models

**DOI:** 10.1242/dmm.050798

**Published:** 2024-08-13

**Authors:** Naghmana Ashraf, Jeanine L. Van Nostrand

**Affiliations:** Department of Molecular and Cellular Biology, Baylor College of Medicine, Houston, TX 77030, USA

**Keywords:** AMPK, Cell signaling, Mouse models

## Abstract

AMP-activated protein kinase (AMPK) is an evolutionarily conserved serine/threonine kinase that monitors the cellular energy status to adapt it to the fluctuating nutritional and environmental conditions in an organism. AMPK plays an integral part in a wide array of physiological processes, such as cell growth, autophagy and mitochondrial function, and is implicated in diverse diseases, including cancer, metabolic disorders, cardiovascular diseases and neurodegenerative diseases. AMPK orchestrates many different physiological outcomes by phosphorylating a broad range of downstream substrates. However, the importance of AMPK-mediated regulation of these substrates *in vivo* remains an ongoing area of investigation to better understand its precise role in cellular and metabolic homeostasis. Here, we provide a comprehensive overview of our understanding of the kinase function of AMPK *in vivo*, as uncovered from mouse models that harbor phosphorylation mutations in AMPK substrates. We discuss some of the inherent limitations of these mouse models, highlight the broader implications of these studies for understanding human health and disease, and explore the valuable insights gained that could inform future therapeutic strategies for the treatment of metabolic and non-metabolic disorders.

## Teaser

**Summary:** Knock-in mouse models of AMPK target proteins deepen our understanding of AMPK biology, revealing new insights and complexities that impact related human disease.

## Introduction

The maintenance of cellular homeostasis is essential for organismal survival. Changes in cellular energy levels, such as ATP, dictate responses at the single-cell and whole-organism levels. The major sensor of cellular energy levels is the highly conserved AMP-activated protein kinase (AMPK), which is evolutionarily conserved from early unicellular organisms to mammals (Hardie, 2007). AMPK is a heterotrimeric enzyme composed of a catalytic α subunit and regulatory β and γ subunits (see Box 1 for more on its structure and function). AMPK is activated in response to various circumstances that lower cellular energy levels, including nutritional deprivation (particularly of glucose), hypoxia, and exposure to toxins that block the mitochondrial respiratory chain complex (Hardie et al., 2012a). In response to these triggers, AMPK coordinates various metabolic pathways that regulate the equilibrium between nutrient and energy supply and demand.

**Box 1. AMPK structure and isoforms**

AMP-activated protein kinase (AMPK) is an obligate heterotrimeric enzyme that consists of a catalytic α subunit, and regulatory β and γ subunits (Steinberg and Hardie, 2023). AMPK has several isoforms. In mammals, the two AMPK α catalytic subunits, α1 and α2, are encoded by the genes *Prkaa1* and *Prkaa2*, respectively; its two regulatory β subunits are encoded by *Prkab1* and *Prkab2*; and its three γ subunits, γ1, γ2 and γ3, are encoded by *Prkag1*, *Prkag2 and Prkag3*, respectively (Cheung et al., 2000; Hardie, 2007; Stapleton et al., 1996; Thornton et al., 1998). Although AMPKα1, AMPKβ1 and AMPKγ1 are widely expressed, the expression of other AMPK isoforms is tissue restricted in a species-specific manner. For example, AMPKα2 and AMPKβ2 are more highly expressed in mouse skeletal and cardiac muscle compared to mouse liver and other tissues (Garcia and Shaw, 2017; Ross et al., 2016b). Whether the varying expression patterns of AMPK isoforms occur in response to diverse physiological or pathological conditions is not known, nor is the extent to which the different isoforms influence AMPK biology. In addition to differences in expression, the various AMPK isoforms can also occupy distinct subcellular compartments, with differing substrate availability (Afinanisa et al., 2021). For example, although the AMPKα2 subunit has a nuclear localization signal that promotes its shuttling to the nucleus, the AMPKα1 subunit has a nuclear export sequence. Consequently, it primarily resides in the cytoplasm and only translocates to the nucleus in a circadian manner or during apoptosis (Cheratta et al., 2022; Khan and Frigo, 2017; Lamia et al., 2009). The different AMPK isoforms also have slightly different biochemical characteristics (Ross et al., 2016b). The two catalytic α subunits differ in their AMP and LKB1 responsiveness, with the levels of AMPKα2 activation being considerably higher than those of AMPKα1 when AMP levels are high. Furthermore, the substrate specificities of AMPKα1 and AMPKα2 are influenced by the presence or absence of a hydrophobic side chain (Salt et al., 1998). The β subunits also have differing affinities for glycogen, with AMPKβ2 having a higher affinity, and the γ subunits differ in their response to AMP levels, with AMPKγ1 and AMPKγ2 being more sensitive to lower AMP/ATP ratios (Ross et al., 2016a,b; Willows et al., 2017). These differences in AMPK isoform biology indicate that the phosphorylation potential of a substrate might differ in different tissues and contexts, highlighting the importance of studying these phosphorylation events *in vivo* to understand their role in health.

**Fig. 1.**
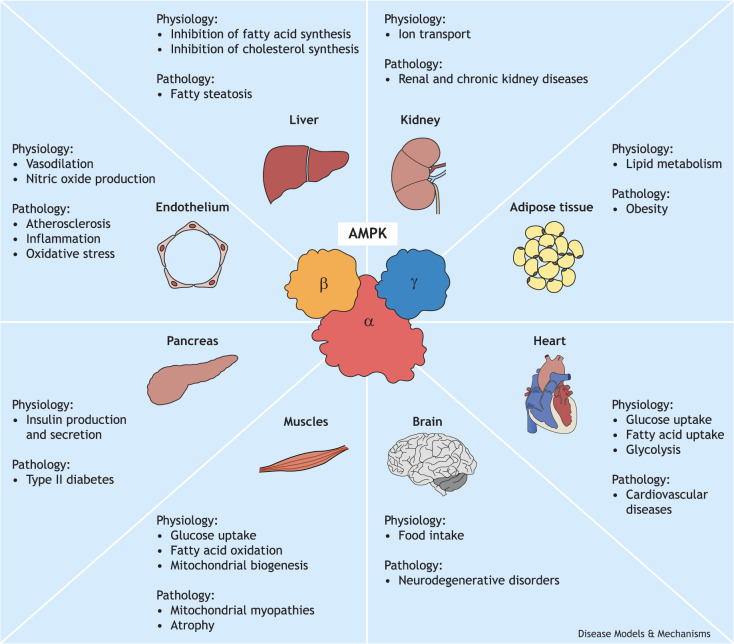
**AMPK in human health and diseases.** The multifaceted involvement of the heterotrimeric protein complex AMP-activated protein kinase (AMPK) in key physiological processes, highlighting its impact on human health and on the development of various diseases. AMPK regulates cellular energy homeostasis, metabolism, stress responses and cell growth, influencing critical functions such as cardiovascular health, neuroprotection and cancer development. Dysregulation of AMPK signaling is implicated in metabolic disorders, neurodegenerative diseases and cancer, highlighting its significance as a potential therapeutic target for a broad spectrum of health conditions.

Given the positive physiological impacts of AMPK activation on metabolism, AMPK is a key target for the prevention and treatment of human disorders, such as cancer and metabolic syndromes (Steinberg and Carling, 2019), and plays a multifaceted role in health and disease (Hardie, 2014; Jeon, 2016; Kim et al., 2016; Li et al., 2017) (Fig. 1). In the muscle, AMPK facilitates glucose uptake, stimulates fatty acid β-oxidation (FAO, see Glossary, Box 2) and promotes mitochondrial biogenesis, thereby enhancing insulin sensitivity and maintaining a healthy body weight (Jørgensen et al., 2021; Lee et al., 2006; Rhein et al., 2021; Thomson, 2018). In the liver, AMPK inhibits fatty acid and cholesterol biosynthesis, induces FAO, and stimulates glucose uptake to oppose fatty liver disease (Box 2) development. This inhibition of fatty liver (Box 2) has been observed both upon treatment of mice with AMPK activators and in the context of constitutively active AMPK expression in the mouse liver (Garcia et al., 2019; Myers et al., 2017; Woods et al., 2017). AMPK also exerts anti-inflammatory and antioxidant effects in the heart and vasculature of mice along with metabolic changes that are crucial for overall vascular health (Rodríguez et al., 2021). As such, the therapeutic targeting of AMPK signaling in humans offers a potential strategy with which to alleviate vascular dysfunction and improve cardiovascular health. However, the role of AMPK in cardiac health is pleiotropic – AMPK hyperactivation can also induce cardiac hypertrophy in mice and humans (Myers et al., 2017; Arad et al., 2003; Li et al., 2019; Kim et al., 2014) – and it has a complex involvement in pulmonary hypertension (Zhao et al., 2021), as both inhibition and activation of AMPK in the lung can be protective. A role for AMPK has also been implicated in the innate immune system (Prantner et al., 2017). AMPK signaling can restrict the replication of multiple viruses, including the hepatitis C virus, in mice and in human cell lines, although how it does so remains poorly understood (Mankouri et al., 2010; Xie et al., 2015, 2016).

**Box 2. Glossary**

**5-aminoimidazole-4-carboxamide-1-β-D-ribofuranoside (AICAR):** AMP mimetic that activates AMPK.

**Anabolism:** metabolic processes involved in building molecules.

**Angiotensin:** hormone that regulates blood pressure by constricting blood vessels.

**Catabolism:** metabolic processes involved in breaking down molecules.

***De novo* lipogenesis:** conversion of dietary carbohydrates into fat.

**Dense granule:** membrane-bound compartment that stores small molecules; release promotes platelet aggregation to form a blood clot.

**Fatty acid β-oxidation (FAO):** process by which fat is broken down to produce energy.

**Fatty acid metabolism:** interconnected pathways for fat synthesis and breakdown.

**Fatty liver:** buildup of fat in the liver.

**Fatty liver disease:** diseases caused by excess buildup of fat in the liver.

**Fibrosis:** excessive scar tissue formation that replaces normal tissue.

**Glycolysis:** metabolic pathway that converts glucose to pyruvate for energy production and macromolecule biosynthesis.

**Glycolytic flux:** measure of the maximum rate of conversion to pyruvate or lactate in cells.

**Innate immunity:** first line of defense by the host that is intended to prevent infection and attack the invading pathogen.

**Ketone bodies:** molecules produced from breakdown of fat during periods of caloric restriction.

**Liposaccharides:** large molecules consisting of a lipid and a polysaccharide that are bacterial toxins.

**Mass spectrometry:** analytical tool for measuring the mass-to-charge ratio; used to identify proteins, metabolites and other molecules.

**Metabolic dysfunction–associated steatotic liver disease:** fatty liver disease not caused by heavy alcohol use. Previously known as non-alcoholic fatty liver disease.

**Metformin:** front-line treatment for diabetes that inhibits mitochondria and activates AMPK.

**Mitochondrial antiviral signaling protein (MAVS):** acts as a switch in the immune signal transduction response to viral infection.

**Myocardial:** heart muscle related.

**Nephropathy:** deterioration of kidney function.

**Orexigenic signaling:** appetite stimulation.

**Renin–angiotensin system (RAS):** regulation of kidney, heart and vasculature physiology.

**Stimulator of interferon genes (STING)–cyclic GMP-AMP synthase (c-GAS) pathway:** senses various pathogens and triggers innate immunity pathways.

**Thrombosis:** blood clot within the blood vessel that limits the flow of blood.

**Triglycerides:** type of fat stored in fat cells.

**Tubulointerstitial fibrosis:** excessive scar tissue formation in the kidney leading to kidney failure.

**Vasoconstriction:** narrowing of blood vessels.

When its functions in health become dysregulated, AMPK can also play an active role in initiating or advancing human pathologies and diseases, including atherosclerosis, diabetes, cancer, neurodegenerative disorders, inflammatory alterations and viral infections (Carling, 2017; Hardie, 2014; Hardie et al., 2012b; Jeon, 2016; Kim et al., 2016; Li et al., 2017). As our understanding of the intricate regulatory mechanisms of AMPK grows, so does its potential as a therapeutic target in various diseases. Harnessing its power to restore metabolic balance while avoiding potential pitfalls is a burgeoning area of research with significant implications for health and disease management.

In this Review, we discuss the successful generation of knock-in phosphorylation-mutant mouse models, highlighting their contributions to studying the *in vivo* consequences of AMPK phosphorylation and deepening our understanding of metabolic homeostasis (summarized in Table 1)*.* These mouse models have not only confirmed the anticipated roles of AMPK signaling in metabolic ailments, such as fatty liver disease and liver cancer, but have also uncovered its involvement in non-metabolic conditions such as thrombosis (Box 2), the anti-viral response and pulmonary hypertension. Alongside offering distinct benefits for investigating AMPK signaling, these mice have also unveiled certain challenges linked with complex mouse models. These challenges, along with the limitations for extrapolating these findings to human pathology, will be further examined in the context of their potential for future research.

**Table 1.**
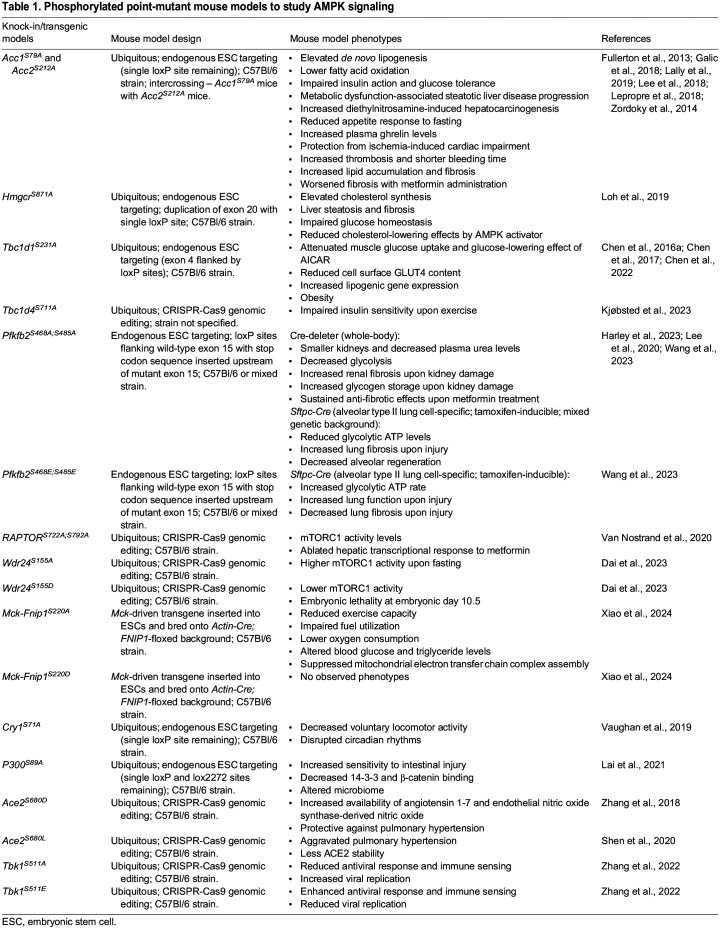
Phosphorylated point-mutant mouse models to study AMPK signaling

## An overview of the physiological and metabolic roles of AMPK

**Fig. 2.**
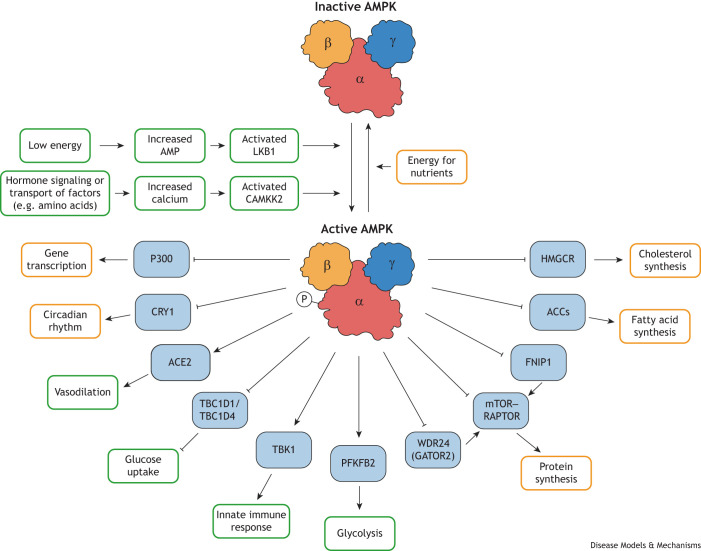
**AMPK activation and downstream signaling pathways.** A schematic of upstream regulation of AMP-activated protein kinase (AMPK) and the numerous signaling cascades initiated upon AMPK activation, highlighting the downstream signaling pathways characterized by knock-in phosphorylation-mutant mouse models. AMPK activation via phosphorylation (‘P’) and energetic stress promotes (green boxes) catabolic pathways for ATP generation while inhibiting (orange boxes) energy-consuming anabolic pathways, ensuring precise maintenance of cellular energy levels. Abbreviations: ACCs, acetyl coenzyme A carboxylases; ACE2, angiotensin-converting enzyme 2; AMP, adenosine monophosphate; CAMKK2, calcium/calmodulin-dependent protein kinase kinase 2; CRY1, circadian cryptochrome-1; FNIP1, folliculin-interacting protein 1; GATOR2, GTPase-activating protein towards Rags 2 protein complex; HMGCR, hydroxy-3-methylglutaryl-coenzyme A reductase; LKB1, liver kinase B1; mTOR, mechanistic target of rapamycin; P300, histone acetyltransferase P300; PFKFB2, 6-phosphofructo-2-kinase/fructose-2,6-bisphosphatase 2; RAPTOR, regulatory-associated protein of mTOR; TBC1D1/TBC1D4, TBC1 domain family members 1 and 4; TBK1, TANK-binding kinase 1; WDR24, WD repeat-containing protein 24.

Cells constantly adjust their metabolic activities to meet energy demands and to respond to fluctuations in nutrient availability caused by physiological stress or pharmacological intervention. When energy is scarce, cellular AMP levels increase, leading to AMPK activation. LKB1 (also known as STK11) is an upstream kinase that activates AMPK in response to increased AMP. CAMKK2 can also activate AMPK in response to increased calcium levels caused by hormone signaling and upon transport of other circulatory factors, such as amino acids, glucose and liposaccharides (Box 2). Once activated, AMPK phosphorylates specific downstream targets to enhance ATP production and reduce ATP consumption (Herzig and Shaw, 2017). AMPK, as a serine/threonine kinase, can directly phosphorylate several substrates, leading to their activation or inhibition (Fig. 2). Over the last decade, the discovery of numerous new AMPK targets has enriched our understanding of the essential processes required to shift cellular metabolism from building molecules (anabolism, Box 2) to breaking them down (catabolism, Box 2). This energy shift regulates cellular growth, as well as various other processes, such as lipid and glucose metabolism, and autophagy. Additionally, AMPK is essential for mitochondrial health, with numerous recently identified AMPK targets implicated in different aspects of maintaining mitochondrial function, including biogenesis, fusion, fission and mitophagy (Herzig and Shaw, 2017; Malik et al., 2023).

The specific combination and subcellular localization of AMPK isoforms and complexes govern their association with upstream and downstream cellular processes (see Box 1). The activation of AMPK pools is contingent upon the type and severity of energetic stress (Zong et al., 2019). Low levels of glucose can activate lysosomal pools of AMPK without changing AMP levels, whereas severe nutrient stress or mitochondrial damage can cause high levels of AMP and activate mitochondrial pools of AMPK (Zong et al., 2019). The localization and activation of AMPK then dictate which subset of downstream targets AMPK can interact with and phosphorylate to determine the biological output. For instance, AMPK activation in mitochondria can lead to the phosphorylation of folliculin-interacting protein 1 (FNIP1) to drive mitochondrial metabolism (Xiao et al., 2024). Additionally, AMPK localization to the lysosome is critical for its ability to inhibit the mechanistic target of rapamycin (mTOR) complex 1 (mTORC1) signaling during energy stress (Carroll and Dunlop, 2017; Morrison et al., 2022). In all, the subcellular localization of AMPK and its downstream substrates can lend an additional layer of regulation to AMPK function that is largely underexplored.

Numerous studies using whole-body and/or conditional AMPK knockout mouse models or tissue-specific AMPK-transgenic mice have demonstrated the role of AMPK regulation in metabolism (Day et al., 2017; Fang et al., 2022; Göransson et al., 2023; Kjøbsted et al., 2018; Muraleedharan and Dasgupta, 2022; Steinberg and Hardie, 2023; Trefts and Shaw, 2021; van der Vaart et al., 2021; Viollet and Foretz, 2016; Viollet et al., 2009a). Additionally, AMPK knock-in mouse models that carry mutations in specific sites or regulatory domains have provided us with insights into AMPK activation, substrate specificity and subcellular localization. However, despite their utility, AMPK mutant mouse models have not, as yet, pinpointed the critical downstream signaling events for AMPK function due to the complexity of AMPK signaling networks and their crosstalk with other pathways.

Much of the research that has been done to better understand AMPK-mediated regulation of individual downstream targets has relied on the use of *in vitro* cell line models or knockout mouse models, making it challenging to attribute precise functions to AMPK activity *in vivo*. When AMPK substrates are knocked out in mouse models, for instance, their loss affects other signaling pathways that also use these targets or alters downstream pathways constitutively, even in the absence of AMPK activation. To overcome these limitations, knock-in mouse models that harbor mutations in the phosphorylation sites of select AMPK substrates have been engineered over the past decade. The generation of such phosphorylation-mutant models has greatly expanded our understanding of the distinct and critical roles of AMPK in the control of both energy metabolism and health.

## Knock-in mouse models to explore AMPK-mediated regulation of metabolic pathways

### AMPK regulation of acetyl-CoA carboxylase in different physiological contexts

**Fig. 3.**
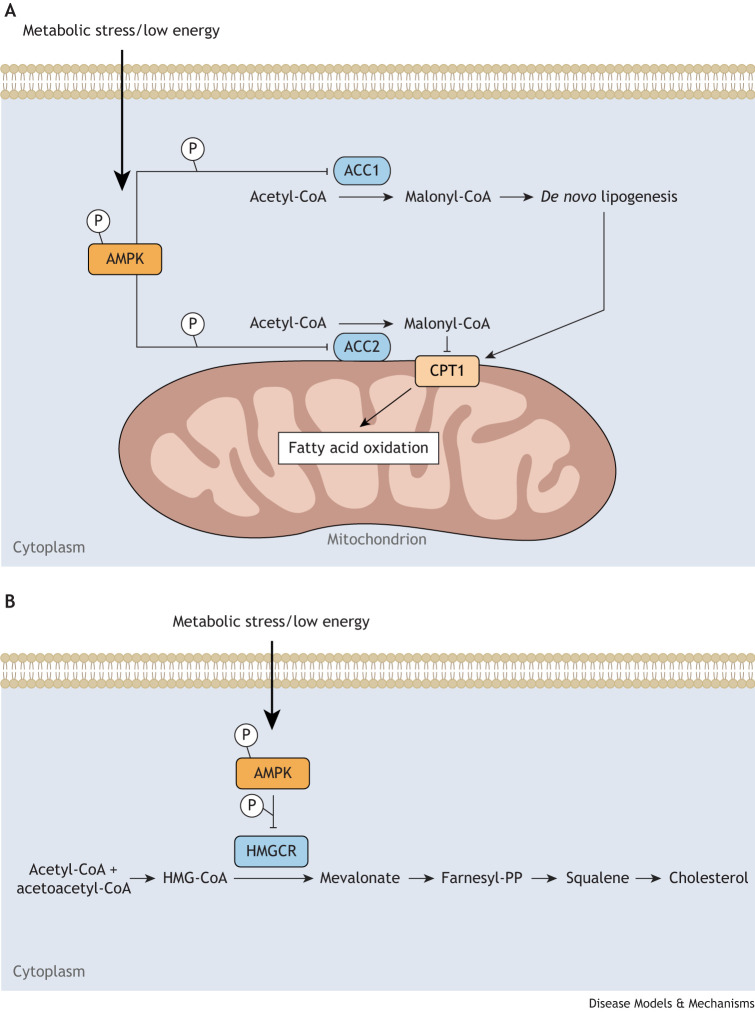
**AMPK–ACC-mediated fatty acid and cholesterol metabolism.** (A) The diagram illustrates the role of AMP-activated protein kinase (AMPK) in regulating fatty acid metabolism through the inhibition of acetyl coenzyme A (CoA) carboxylases 1 (ACC1) and 2 (ACC2). Upon activation, AMPK phosphorylates and inhibits ACCs, leading to decreased production of malonyl-CoA and reduced fatty acid synthesis. Simultaneously, AMPK activation enhances fatty acid oxidation by promoting the activity of enzymes involved in mitochondrial β-oxidation. Together, these actions contribute to AMPK-mediated regulation of fatty acid metabolism and various pathological outcomes. (B) The diagram illustrates the role of AMPK in regulating cholesterol metabolism through the phosphorylation and inhibition of hydroxy-3-methylglutaryl-coenzyme A reductase (HMGCR), the rate-limiting enzyme in cholesterol synthesis. This inhibition leads to reduced cholesterol biosynthesis, thereby modulating cellular cholesterol levels and contributing to cholesterol homeostasis. Abbreviations: CPT1, carnitine palmitoyltransferase 1; farnesyl-PP, farnesyl diphosphate; HMG-CoA, hydroxy-3-methylglutaryl-coenzyme A.

Acetyl coenzyme A (CoA) carboxylases (ACCs) are key regulators in fatty acid metabolism (Box 2), catalyzing the conversion of acetyl-CoA into malonyl-CoA, an essential precursor for fatty acid biosynthesis (Fig. 3A). In mammals, ACC1 and ACC2, encoded by the *ACACA* and *ACACB* genes, respectively, produce malonyl-CoA in different subcellular locations, leading to distinct biological effects. ACC1 (also known as ACACA), found in the cytosol, controls malonyl-CoA biosynthesis necessary for *de novo* lipogenesis (Box 2), whereas ACC2 (ACACB), located at the outer mitochondrial membrane, generates malonyl-CoA to obstruct fatty acid transport into the mitochondria, thereby inhibiting FAO. Consequently, ACCs are critical rate-controlling enzymes in malonyl-CoA synthesis and are potent suppressors of mitochondrial FAO (Brownsey et al., 2006; Steinberg and Kemp, 2009) (Fig. 3A).

ACCs are regulated in various ways, including via their activation by citrate and via their phosphorylation at different sites by various protein kinases, including AMPK (Carling et al., 1987; Hardie and Guy, 1980; Munday et al., 1986; Tipper and Witters, 1982). AMPK is an essential kinase for ACC1 and ACC2 and phosphorylates ACCs at specific serine residues (serine 79 in ACC1 and serine 212 in ACC2), inhibiting their enzymatic activity (Hardie, 2004). Phosphorylation at these residues provides a reliable indicator of AMPK activation in cellular and tissue contexts (Fullerton et al., 2013; Munday et al., 1988; Scott et al., 2002). The AMPK-mediated inhibition of ACC activity reduces malonyl-CoA levels, resulting in decreased triglyceride (Box 2) synthesis and increased β-oxidation in cells (Saggerson, 2008; Srivastava et al., 2012). This AMPK-mediated inhibition of ACC activity was first exemplified by the observed decline in plasma triglyceride levels during 5-aminoimidazole-4-carboxamide ribonucleotide (AICAR, Box 2) infusion in lean and obese rodents (Muoio et al., 1999). It is also evident when AMPKα2 (encoded by *Prkaa2*) is overexpressed in the liver of mice, resulting in reduced plasma triglyceride levels and increased plasma ketone bodies (Box 2), which are indicative of elevated hepatic β-oxidation (Foretz et al., 2005). Conversely, when liver-specific *Prkaa2* was deleted in mice, it resulted in increased plasma triglyceride levels and a decrease in plasma ketone bodies (Andreelli et al., 2006), emphasizing the key role of AMPK in balancing hepatic lipogenesis and β-oxidation.

To investigate the physiological impact of these phosphorylation events and to provide mechanistic insights into the effects of AMPK regulation of ACCs, Fullerton et al. (2013) investigated mice that carried alanine mutations in the serine 79 and serine 212 phosphorylation sites of ACC1 and ACC2, respectively. These mice revealed that AMPK-mediated inhibitory phosphorylation of ACC1 and ACC2 at these residues is crucial for maintaining lipid homeostasis and for the beneficial effects of metformin (Box 2) on insulin sensitivity. Double knock-in mice (*Acc1^S79A^;Acc2^S212A^*, i.e. *Acc^DKI^*) carrying both of these mutations showed elevated lipogenesis, lower FAO and metabolic dysfunction-associated steatotic liver disease (Box 2) progression. They also exhibited impaired insulin action and glucose tolerance. *Acc^DKI^* mice also became obese on a high-fat diet, similar to wild-type mice, but were resistant to the hypolipidemic and insulin-responsive effects of metformin that can be observed in wild-type obese mice (Fullerton et al., 2013).

ACC-derived fatty acids have been indirectly associated with platelet activation. As such, *Acc^DKI^* mice have also been used to investigate ACC regulation in platelets. Notably, *Acc^DKI^* mice showed increased thrombosis and shorter bleeding time, and *Acc^DKI^* platelets had altered phospholipid content and increased dense granule (Box 2) release, which affected thrombus formation, implicating impaired AMPK–ACC signaling in the observed amplified thrombosis (Lepropre et al., 2018). Thus, *Acc^DKI^* platelets have a gain-of-function effect on thrombus formation and potentially impact hemostasis, which can contribute to cardiovascular diseases, thrombotic disorders, bleeding disorders and complications in conditions such as cancer, sepsis and autoimmune disorders.

Surprisingly, the AMPK-dependent phosphorylation of ACCs is considered to be nonessential for sustaining myocardial (Box 2) FAO rates and, consequently, cardiac function. Although *Acc^DKI^* mice show increased levels of malonyl-CoA, a key regulator of FAO, they have no discernable differences in myocardial FAO rates compared to those in wild-type mice. It remains possible that the prolonged disruption of AMPK signaling to ACC1 and ACC2 in these mice results in the development of compensatory mechanisms that influence these outcomes. That said, the loss of AMPK-mediated inhibitory ACC phosphorylation did not impact cardiac morphology or function in *Acc^DKI^* mice under baseline or increased workload conditions (Zordoky et al., 2014). Interestingly, the *Acc^DKI^* mice did demonstrate improved recovery of cardiac function after ischemia, indicating that the loss of ACC inhibition by AMPK is protective against ischemia-induced functional impairment in the heart, although the reason for this improvement needs further investigation (Zordoky et al., 2014).

In the mouse kidney, ACC phosphorylation by AMPK is essential for the anti-fibrotic effect of metformin. In wild-type mice, upon induction of folic acid-induced nephropathy (Box 2) (FAN), the administration of metformin is linked to a decrease in fibrosis (Box 2) and lipid accumulation. However, this protective effect of metformin on FAN is lost in *Acc^DKI^* mice; instead, *Acc^DKI^* mice exhibit even worse fibrosis and increased lipid accumulation upon metformin treatment compared to untreated mice. These findings corroborate reported observations that the phosphorylation of ACCs by AMPK is reduced in tubular epithelial cells in mice with FAN, indicating that impaired ACC phosphorylation in renal injury plays a role in the onset of tubulointerstitial fibrosis (Box 2) (Lee et al., 2018).

AMPK phosphorylation of ACCs is also required for the augmentation of appetite during metabolic stress and during orexigenic signaling (Box 2), which is induced in response to fasting, exposure to cold and ghrelin (GHRL; hunger hormone) stimulation. *Acc^DKI^* mice show reduced appetite and impaired orexigenic responses to ghrelin, indicating that these mice have a ghrelin signaling defect. Accordingly, therapeutic strategies that target ACC phosphorylation might be able to suppress appetite in response to metabolic stress, which can occur in conditions such as diabetes and obesity. *Acc^DKI^* mice also displayed reduced locomotor activity; however, this reduction was considered independent of ghrelin signaling as wild-type mice treated with a ghrelin receptor antagonist did not phenocopy *Acc^DKI^* mice (Galic et al., 2018). In contrast, *Acc^DKI^* mice are not different from wild-type mice in their response to thermogenesis and to leptin (LEP)-induced suppression of food intake, which suggests that the AMPK-mediated phosphorylation of ACCs is dispensable for these processes.

The role of ACCs in cancer is receiving increased interest due to their involvement in lipid metabolism, providing cancer cells with essential building blocks for rapid proliferation and survival, particularly in lipogenic cancers such as breast, prostate and liver cancer. As such, the ability of AMPK to regulate ACCs is an attractive therapeutic avenue. Specifically, *Acc^DKI^* mice injected with diethylnitrosamine to induce hepatocellular carcinoma show increased liver *de novo* lipogenesis and worse outcomes, such as an increased number of liver lesions, compared to wild-type mice (Lally et al., 2019). Similarly, human liver cancer cells (HepG2) with the *ACC1^S80A^* knock-in mutation, equivalent to that in *Acc1^S79A^* mice, show increased *de novo* lipogenesis and proliferation (Lally et al., 2019). Furthermore, small-molecule therapies that mimic ACC phosphorylation, such as ND-654 and ND-646, have been shown to prevent and treat hepatocellular carcinoma in rat models and to prevent non-small-cell lung cancer development in human cells and in mice (Lally et al., 2019; Svensson et al., 2016). Thus, studies using *Acc^DKI^* mice have uncovered the importance of the AMPK-mediated phosphorylation of ACCs for fulfilling various AMPK-dependent functions *in vivo* that extend across the spectrum of metabolic regulation and energy homeostasis. Studies using *Acc^DKI^* mice continue to be instrumental in deciphering the intricate, yet finely tuned, mechanisms regulated by AMPK in the orchestration of fatty acid metabolism. In addition to regulating fatty acid biosynthesis and oxidation, AMPK also plays an important role in regulating the use of fatty acids for lipid and cholesterol metabolism.

### HMGCR in AMPK-mediated cholesterol metabolism

AMPK regulates lipid metabolism pathways, particularly in the liver, where AMPK plays a pivotal role in managing overall energy levels by controlling fatty acid synthesis and carbohydrate storage and release. In the mammalian liver, AMPK coordinates the activity of enzymes involved in lipid metabolism, thereby regulating the allocation of fatty acids among oxidative and biosynthetic pathways (Fang et al., 2022; Viollet et al., 2006). During cholesterol synthesis, AMPK phosphorylates and inhibits hydroxy-3-methylglutaryl-CoA (HMG-CoA) reductase (HMGCR), thereby blocking the rate-limiting step of HMG-CoA conversion into mevalonate, thus preventing cholesterol biosynthesis (Carling et al., 1987; Friesen and Rodwell, 2004; Viollet et al., 2009b) (Fig. 3B). Both total and liver-specific *Prkaa2* knockout mice exhibited elevated plasma levels of total and high-density lipoprotein (‘good’) cholesterol, although not in a manner that was statistically distinct from controls (Andreelli et al., 2006; Viollet et al., 2003). When the *Hmgcr* gene was deleted in mice, they did not survive past day 3 of early embryonic development (Ohashi et al., 2003). In addition, liver function significantly deteriorated in mice with a liver-specific HMGCR deficiency, and these mice did not survive beyond 6 weeks (Nagashima et al., 2012). These findings underscore the essential role of the mevalonate pathway in mouse development and for their overall survival.

The phosphorylation of HMGCR at serine 871 by AMPK inhibits HMGCR activity and suppresses cholesterol synthesis under conditions of depleted ATP levels. However, mutation of serine 871 to alanine does not impact the response of HMGCR activity to feedback control mechanisms induced by high levels of downstream products, such as mevalonate or low-density lipoproteins *in vitro* (Clarke and Hardie, 1990; Sato et al., 1993). This finding raises questions about the significance of AMPK–HMGCR signaling in the broader context of the whole organism, particularly outside situations that are characterized by severe metabolic stress.

To assess the biological significance of the AMPK-dependent phosphorylation of HMGCR, Loh et al. (2019) created knock-in mice with a serine 871 to alanine mutation in the *Hmgcr* gene (*Hmgcr^S871A^*). This knock-in mutation abrogated the AMPK-mediated phosphorylation of HMGCR and thus inhibited AMPK–HMGCR signaling. When *Hmgcr^S871A^* mice were placed under conditions of metabolic stress, such as high-carbohydrate feeding, they exhibited elevated cholesterol synthesis, liver steatosis and fibrosis, and impaired glucose homeostasis. Furthermore, hepatocytes from these mice showed decreased responsiveness to the cholesterol-lowering effects of AMPK activators, A769662 and AICAR. Thus, the inhibition of HMGCR by AMPK is crucial for cholesterol homeostasis. However, the physiological contexts in which this inhibition is important remain to be explored, and *Hmgcr^S871A^* mice provide a robust and practical model to systematically define and characterize these contexts. Analysis of the role of AMPK in regulating fatty acid and lipid metabolism must also take into account its role in glucose metabolism.

### TBC1D1 and TBC1D4 in AMPK-mediated glucose metabolism

**Fig. 4.**
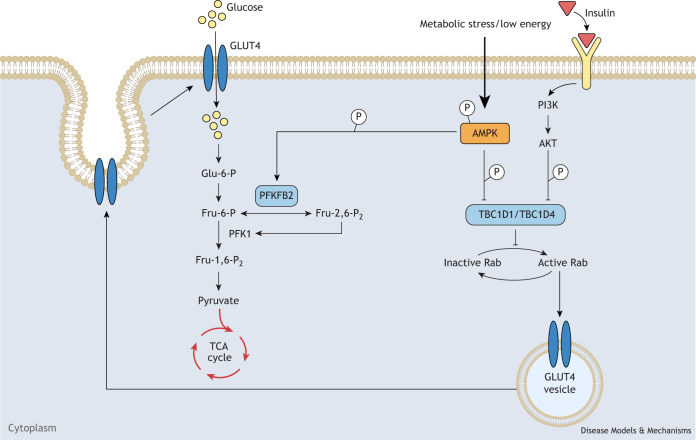
**AMPK-mediated glucose metabolism.** The diagram depicts AMP-activated protein kinase (AMPK) regulation of glucose metabolism through TBC1D1/TBC1D4 and PFKFB2. Insulin stimulation or AMPK activation phosphorylates and inhibits TBC1D1 and/or TBC1D4, leading to increased translocation of glucose transporter type 4 (GLUT4)-containing vesicles to the plasma membrane and induced glucose uptake into cells. Additionally, AMPK phosphorylates and modulates PFKFB2 activity, which results in altered levels of fructose-2,6-bisphosphate (Fru-2,6-P_2_) and glycolytic flux. Together, these phosphorylation events contribute to the modulation of cellular glucose metabolism by AMPK. Abbreviations: AKT, α serine/threonine protein kinase; Fru-1,6-P_2_, fructose-1,6-bisphosphate; Fru-6-P, fructose-6-phosphate; Glu-6-P, glucose-6-phosphate; PFK1, phosphofructo kinase 1; PFKFB2, 6-phosphofructo-2-kinase/fructose-2,6-bisphosphatase 2; PI3K, phosphoinositide 3-kinase; TBC1D1/TBC1D4, TBC1 domain family members 1 and 4; TCA cycle, tricarboxylic acid cycle.

Insulin and exercise are important stimuli for increasing skeletal muscle glucose transport, and both trigger the redistribution of GLUT4 (also known as SLC2A4) glucose transporters to the cell surface membranes. A solitary bout of exercise can elicit enhanced insulin-stimulated glucose transport in muscles and improve overall insulin sensitivity throughout the body in both humans and mice. TBC1D4 (also known as AKT substrate of 160 kDa or AS160) and TBC1D1 are Rab GTPase-activating proteins, which are involved in the regulation of muscle glucose transport triggered by insulin and/or exercise. TBC1D1 and TBC1D4 dysregulation is associated with type 2 diabetes mellitus, contributing to insulin resistance and impaired glucose metabolism (Mafakheri et al., 2018). TBC1D4 and TBC1D1 both inhibit GLUT4 translocation in the basal state. In response to insulin or exercise, TBC1D4 and TBC1D1 are phosphorylated, which leads to binding by 14-3-3 proteins and subsequent inhibition. AMPK phosphorylates both TBC1D4 and TBC1D1 at multiple sites to modulate their activity, thereby regulating glucose transport and subsequent cellular functions related to glucose metabolism and GLUT4 translocation (Fig. 4). Notably, TBC1D1 deficiency causes decreased GLUT4 expression levels in mouse skeletal muscle, and TBC1D1 is thought to mediate AMPK-governed glucose homeostasis, GLUT4 trafficking and muscle glucose uptake in a context-dependent manner (Chen et al., 2008; Chen et al., 2011a,b; Eickelschulte et al., 2021; Geraghty et al., 2007; Pehmøller et al., 2009; Ramm et al., 2006; Richter and Hargreaves, 2013).

To further investigate the regulation of TBC1D1 by AMPK, Chen et al. (2017) created a serine 231 to alanine (*Tbc1d1^S231A^*) knock-in mouse model. The researchers observed that the glucose-lowering effect of AICAR, a known AMPK-activating agent, was attenuated in *Tbc1d1^S231A^* mice, which showed impaired muscle glucose uptake and reduced GLUT4 content at the cell surface. However, *Tbc1d1^S231A^* mice exhibited no impairment in exercise-induced muscle glucose uptake nor in exercise capacity (Chen et al., 2017). Instead, they developed obesity on a chow diet relative to wild-type mice, which was caused by the hypersecretion of insulin-like growth factor-1 (IGF1) and by increased lipogenic gene expression downstream of AKT and mTOR (Chen et al., 2016a). In aged *Tbc1d1^S231A^* mice (18 months old), the researchers found high hepatic triglyceride levels and the activation of peroxisome proliferator-activated receptor γ (PPARγ or PPARG) signaling, which is a transcription factor responsible for the expression of lipogenic genes and regulation of fatty acid storage and glucose metabolism (Chen et al., 2022). Thus, these mice revealed a novel regulatory mechanism that links energy status to the development of obesity through the control of IGF1 secretion and PPARγ signaling. Targeting the AMPK–TBC1D1 signaling nexus might have therapeutic implications for the treatment of obesity and metabolic syndrome.

TBC1D4 is phosphorylated at serine 711 both in response to insulin stimulation and AMPK activation during exercise and muscle contraction (Treebak et al., 2010). AMPK is crucial for enhancing whole-body and muscle insulin sensitivity during exercise and muscle contraction, with TBC1D4 playing a pivotal role in this process (Kjøbsted et al., 2017). Kjøbsted et al. (2023) investigated the role of serine 711 phosphorylation in TBC1D4 in improving insulin sensitivity in skeletal muscle after exercise and contraction using a knock-in mouse model with a serine 711 to alanine mutation (*Tbc1d4^S711A^*). The authors observed no effect on body weight, blood glucose levels or muscle glucose uptake in these knock-in mice. However, female *Tbc1d4^S711A^* mice failed to improve insulin sensitivity in response to exercise in comparison to wild-type mice, which was associated with decreased TBC1D4 mRNA and protein expression. This failure in muscle insulin sensitivity was blunted in male *Tbc1d4^S711A^* mice. Notably, *Tbc1d4* heterozygous knockout mice (*Tbc1d4^+/−^*), which have similarly reduced levels of TBC1D4 protein expression, exhibit improved insulin sensitivity upon exercise, indicating that reduced TBC1D4 expression levels were not responsible for the lack of improvement in muscle insulin sensitivity in the female *Tbc1d4^S711A^* mice (Kjøbsted et al., 2023). These results indicate that AMPK-dependent phosphorylation of TBC1D4 serine 711 is essential for enhancing insulin sensitivity after exercise. Beyond fatty acid metabolism and glucose uptake, glucose utilization is another regulatory target of AMPK important for cellular metabolism.

### PFKFB2 in AMPK-mediated glycolysis

In addition to driving increases in glucose uptake, AMPK also stimulates glycolytic flux (Box 2) to raise ATP levels. AMPK phosphorylates 6-phosphofructo-2-kinase/fructose-2,6-bisphosphatase 2 (PFKFB2), the enzyme that makes and breaks down fructose-2,6-bisphosphate (Fru-2,6-P_2_) during glycolysis (Box 2). Phosphorylation of serine 466 in PFKFB2 by AMPK increases its kinase activity, thus promoting glycolytic flux in humans and mice (Marsin et al., 2000) (Fig. 4). PFKFB2 can also be phosphorylated on serine 483 by other kinases, including the protein kinase A (PKA) and protein kinase B (PKB, also known as AKT) family, which mediates PFKFB2 activity and glycolysis stimulated by growth factors (Bertrand et al., 1999; Rubio et al., 2003).

To investigate the role of AMPK-dependent phosphorylation of PFKFB2, Lee et al. (2020) generated mice harboring serine to alanine mutations at both serine 468 and serine 485 (serine 466 and serine 483 in the human protein) to prevent phosphorylation and activation of PFKFB2 (*Pfkfb2^S468A;S485A^*). Analysis of homozygous *Pfkfb2^S468A;S485A^* mice revealed smaller kidneys and decreased plasma urea levels. This was associated with a decrease in glycolysis and PFKFB2 protein levels in mouse kidney cells derived from *Pfkfb2^S468A;S485A^* mice compared to those in controls. In models of kidney disease, *Pfkfb2^S468A;S485A^* mice exhibited higher levels of renal fibrosis upon unilateral ureteric obstruction and increased glycogen storage upon FAN (Lee et al., 2020). Interestingly though, the anti-fibrotic effects of metformin were not prevented in *Pfkfb2^S468A;S485A^* mice, suggesting that AMPK-mediated regulation of fibrosis is not solely due to phosphorylation of PFKFB2 and its subsequent induction of glycolysis (Harley et al., 2023).

Subsequent analysis of PFKFB2 serine to alanine mutations in mouse lungs revealed a critical role for phosphorylation in alveolar regeneration upon lung injury. Wang et al. (2023) used an alveolar type II (AT2) cell-specific Cre (*Sftpc-CreER*) to investigate the consequence of loss of PFKFB2 regulation using both *Sftpc-CreER;Pfkfb2^S468A;S485A^* mice and mice harboring glutamic acid (phosphomimetic) mutations at these sites to mimic the phosphorylated state (*Sftpc-CreER;Pfkfb2^S468E;S485E^*). Analysis of AT2 cells from these mice revealed reduced and increased ATP levels derived from glycolysis in *Pfkfb2^S468A;S485A^* and *Pfkfb2^S468E;S485E^* cells, respectively. Upon lung injury, *Sftpc-CreER;Pfkfb2^S468A;S485A^* mice exhibited a reduction in newly differentiated alveolar type I (AT1) cells derived from AT2 cells, increased lung fibrosis and decreased lung function relative to control mice. In contrast, *Sftpc-CreER;Pfkfb2^S468E;S485E^* mice displayed decreased lung fibrosis and improved lung function relative to control mice (Wang et al., 2023). Overall, these results demonstrate a role for PFKFB2 phosphorylation in the regulation of glycolytic activity and its importance in injury response, which can, at least partially, be attributed to AMPK function.

In all, these studies using knock-in mouse models of metabolic enzymes have begun to define the role of AMPK in glucose, lipid and fatty acid metabolic signaling pathways. Observations from these mice have further revealed physiological and pathological conditions in which AMPK-dependent phosphorylation of metabolic enzymes plays critical functions. However, the influence of AMPK on metabolism extends beyond regulation at the enzyme level, encompassing regulation of other metabolic master regulators such as mTOR.

## Knock-in mouse models to explore AMPK-mediated inhibition of mTOR

**Fig. 5.**
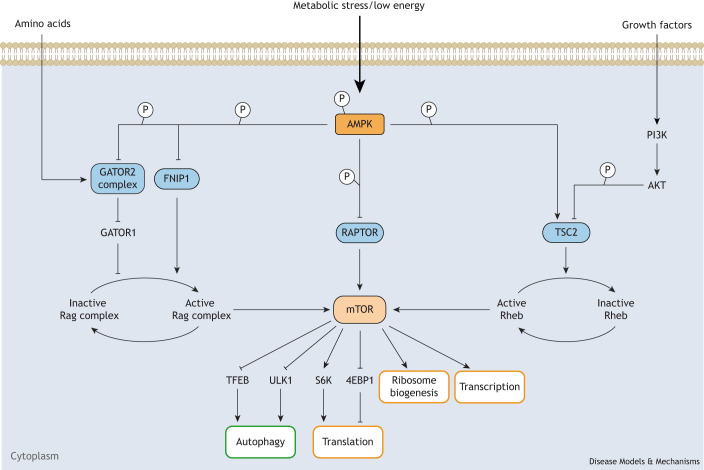
**AMPK-mediated mTOR regulation.** The diagram illustrates the role of AMP-activated protein kinase (AMPK) in inhibiting the mechanistic target of rapamycin (mTOR) signaling pathway under various stimuli to upregulate (green box) or downregulate (orange boxes) downstream metabolic processes. Upon activation, AMPK phosphorylates and inhibits the GATOR2 complex and folliculin-interacting protein 1 (FNIP1), which both induce mTOR activity by promoting guanine nucleotide exchange on the Rag complex. AMPK also phosphorylates and inhibits RAPTOR, which is an obligate partner required for mTOR complex 1 (mTORC1) activity. In opposition of PI3K/AKT signaling, AMPK phosphorylates and activates TSC2, which inhibits mTOR signaling by promoting the conversion of Rheb-GTP to Rheb-GDP, an inactive form of Rheb. Abbreviations: 4EBP1, eIF4E-binding protein 1; AKT, α serine/threonine-protein kinase; GATOR, GTPase-activating protein towards Rags; PI3K, phosphoinositide 3-kinase; Rag, Ras-related GTP binding protein; RAPTOR, regulatory-associated protein of mTOR; Rheb, Ras homolog enriched in brain; S6K, ribosomal S6 kinase; TFEB, transcription factor EB; TSC2, tuberous sclerosis complex 2; ULK1, Unc-51-like kinase 1.

mTOR is a serine/threonine kinase that regulates cell growth and survival by integrating nutrient and hormonal signals (Panwar et al., 2023; Szwed et al., 2021). mTOR is a highly conserved regulator of cell growth, and its activation relies on positive signals from nutrients (such as glucose and amino acids) as well as growth factors (such as insulin) (Liu and Sabatini, 2020; Wullschleger et al., 2006). mTOR exists in two complexes, mTORC1 and mTORC2, which have distinct substrate specificities and are differentially regulated and sensitive to rapamycin (Linde-Garelli and Rogala, 2023). One of these complexes, mTORC1, is sensitive to nutrient levels and serves as a central regulator of cell growth, angiogenesis and metabolism (Liu and Sabatini, 2020; Sabatini, 2006). mTORC1 comprises four known subunits: mTOR, mLST8 (also known as Gbl), PRAS40 (also known as AKT1S1) and RAPTOR (or RPTOR) (Haar et al., 2007; Sabatini, 2006; Sancak et al., 2007). RAPTOR acts as a scaffold to bring downstream substrates, such as 4E-BP1 (EIF4EBP1) and ribosomal S6 kinase, to the mTORC1 complex (Nojima et al., 2003; Schalm et al., 2003) (Fig. 5).

AMPK and mTOR exert opposing roles in regulating cellular growth in response to fluctuations in energy levels. Whereas AMPK becomes active when nutrients are scarce and inactive when nutrients are abundant, mTOR does the opposite, becoming active when nutrients are abundant and inactive when they are scarce (Wullschleger et al., 2006). This disparate response to energy levels means that AMPK and mTORC1 have competing roles in lipid biosynthesis and autophagy.

### RAPTOR in AMPK-mediated mTORC1 regulation

The antagonistic relationship between AMPK and mTORC1 is in part due to the ability of AMPK to directly inhibit mTORC1 via phosphorylation of RAPTOR to prevent the interaction of RAPTOR with mTOR and cause its sequestration by 14-3-3 (Gwinn et al., 2008). RAPTOR is phosphorylated by AMPK at two independent sites: serine 722 and serine 792 (Gwinn et al., 2008). Van Nostrand et al. (2020) generated a dual phosphorylation mouse model harboring an alanine residue at both AMPK phosphorylation sites in RAPTOR (*Raptor^S722A;S792A^*). An analysis of primary hepatocytes and livers from *Raptor^S722A;S792A^* mice revealed that AMPK-mediated regulation of RAPTOR is required to induce translational and gene expression changes caused by the anti-diabetes drug metformin (Van Nostrand et al., 2020). Notably, the mutation of two AMPK phosphorylation sites in RAPTOR alone was not sufficient to completely prevent the regulation of mTORC1 by AMPK, as the additional deletion of the mTORC1 negative regulator TSC2, another critical substrate of AMPK, was required to completely abrogate mTORC1 function (Howell et al., 2017; Van Nostrand et al., 2020) (Fig. 5). AMPK signaling upon metformin treatment of primary hepatocytes was shown to be necessary for mTORC1 inhibition and to regulate both anabolic and inflammatory programs, two beneficial consequences of the mechanism of action of metformin (Van Nostrand et al., 2020). These results underscore the usefulness of these mice in delineating the connection between AMPK and mTORC1 in multiple contexts. The dysregulation of this relationship has been observed in conditions such as cancer, metabolic disorders, neurodegenerative diseases and cardiovascular diseases (Shackelford et al., 2013; Shaw et al., 2004; Zhou et al., 2013), but the relevance of this dysregulation remains to be understood. These *Raptor^S722A;S792A^* knock-in mutant mice thus provide a tool with which to investigate the causative role of mTORC1 inhibition in AMPK-dependent phenotypes and therapies. In addition to direct phosphorylation of the mTORC1 complex via RAPTOR, AMPK also phosphorylates and inhibits other upstream activators of mTORC1, such as the GATOR2 complex.

### GATOR2 in AMPK-mediated mTORC1 regulation

GTPase-activating protein towards Rags 2 (GATOR2) is a protein complex involved in the activation of mTORC1 signaling pathway (Bar-Peled et al., 2013). This complex consists of several proteins, including Mios, WDR24, WDR59, SEH1L and SEC13 (Kim and Guan, 2019). GATOR2 coordinates cellular responses to nutrient availability and growth signals, thereby regulating processes such as protein synthesis, cell growth and metabolism. Its activity is tightly controlled to ensure proper cellular and organismal adaptation to changing environmental conditions and metabolic demands. The primary function of GATOR2 is to inhibit GATOR1. GATOR1 is a protein complex that inhibits mTORC1 activity by preventing guanine nucleotide exchange on the Rag complex, keeping Rag in an inactive state. When GATOR1 is inactivated, active Rag recruits mTORC1 to the lysosome, where mTORC1 becomes activated (Kim et al., 2008; Sancak et al., 2008). By antagonizing the inhibitory activity of GATOR1, GATOR2 promotes the activation of mTORC1 in the presence of sufficient nutrients and growth factors (Bar-Peled et al., 2013; Khalil et al., 2023; Kim and Guan, 2019) (Fig. 5). Conversely, in response to nutrient deprivation or cellular stress, inhibition of GATOR2 leads to GATOR1 activation and to subsequent mTORC1 inhibition, which promotes autophagy, allowing cells to degrade and recycle damaged or unnecessary cellular components to maintain cellular homeostasis and adapt to stressors (Bar-Peled et al., 2013; Liu and Sabatini, 2020; Panchaud et al., 2013; Sahu and Ben-Sahra, 2023; Wei et al., 2014).

AMPK regulates GATOR2 by phosphorylating the WDR24 component of this complex on serine 155, to suppress anabolic processes and to conserve energy during periods of energy stress (Dai et al., 2023). Dai et al. (2023) generated a *Wdr24* knock-in mouse model, in which serine 155 was replaced with alanine (*Wdr24^S155A^*), to investigate the role of AMPK and the GATOR2 complex in regulating mTORC1 activation in response to glucose availability. These mice lack the AMPK-mediated regulation of WDR24 and show higher mTORC1 activity in response to fasting. An analysis of mouse embryonic fibroblasts from *Wdr24^S155A^* mice revealed that AMPK-dependent phosphorylation of WDR24 at serine 155 suppresses glucose-mediated mTORC1 activation. These researchers also generated knock-in mice with a phosphomimetic WDR24 serine 155 to aspartic acid change (*Wdr24^S155D^*). An analysis of *Wdr24^S155D/S155D^* embryonic liver tissue showed lower mTORC1 activity compared to that in wild-type littermates (Dai et al., 2023). However, homozygous *Wdr24^S155D/S155D^* are small in size and do not survive beyond embryonic day 10.5, with only heterozygous *Wdr24^S155D/+^* mice surviving. These mice reveal a role for GATOR2 in glucose sensing and highlight the importance of balanced control of mTORC1 activity via AMPK and the GATOR2 complex. As a regulatory safeguard, AMPK also inhibits mTORC1 via Rag through a secondary mechanism involving FNIP1.

### FNIP1 in the AMPK-mediated exercise response

FNIP1 is a cytoplasmic protein that functions in various cellular processes, including energy metabolism, autophagy and mitochondrial function (Malik et al., 2023). FNIP1 interacts with folliculin (FLCN), a tumor suppressor protein. FNIP1 induces mTORC1 activity by serving as a GTPase for RagC (RRAGC) and/or RagD (RRAGD) of the Rag complex, switching the Rag complex into an active state (Tsun et al., 2013) and promoting lysosomal localization (Ramirez Reyes et al., 2021) (Fig. 5). As such, FNIP1 is implicated in the regulation of lysosomal function and nutrient sensing. When cellular energy levels are low, activated AMPK phosphorylates FNIP1 at multiple sites, altering its interactions with other proteins and inhibiting its activity (Malik et al., 2023). Indeed, FNIP1 has been found to be an AMPK effector for mitochondrial adaptation to exercise, providing insights into the mechanisms of exercise tolerance in health and disease. Its dysregulation has been associated with various diseases, including cancer and metabolic disorders (de Martín Garrido and Aylett, 2020; Malik et al., 2023; Ramirez Reyes et al., 2021).

Xiao et al. (2024) explored the impact of AMPK-mediated FNIP1 phosphorylation on mitochondrial regulation and exercise physiology by expressing muscle-specific *Fnip1* transgenes in a muscle *Fnip1* knockout mouse strain. The *Fnip1* transgene was either wild-type (*Mck-Fnip1^WT^*) or harbored a single AMPK phosphorylation site mutation: serine 220 to alanine (*Mck-Fnip1^S220A^*; phospho-mutant) or serine 220 to aspartic acid (*Mck-Fnip1^S220D^*; phosphomimetic) (Xiao et al., 2024). No phenotypes were reported in the *Mck-Fnip1^S220D^* mice compared to *Mck-Fnip1^WT^* mice. However, *Mck-Fnip1^S220A^* mice showed reduced exercise performance compared to *Mck-Fnip1^WT^* mice, which was marked by decreased running time and distance, elevated blood lactate levels and decreased exercise tolerance. *Mck-Fnip1^S220A^* mice also showed impaired metabolic substrate utilization, lower oxygen consumption, altered blood glucose and triglyceride levels, and reduced muscle glycogen content. These findings indicate that the phosphorylation of FNIP1 at serine 220 by AMPK has a role in regulating muscle metabolism, linking metabolic flexibility to exercise performance. *Mck-Fnip1^S220A^* mice also exhibited suppressed mitochondrial respiration rates during exercise but normal mTORC1 signaling compared to *Mck-Fnip1^WT^* mice, indicating that the phosphorylation of serine 220 in FNIP1 is crucial for normal mitochondrial function and electron transport chain complex formation in skeletal muscle during exercise (Xiao et al., 2024). These studies demonstrate that the exercise-induced phosphorylation of FNIP1 at serine 220 by AMPK plays a crucial role in modulating the metabolic capacity of mitochondria in skeletal muscle during exercise.

Overall, these studies using knock-in mouse models targeting AMPK phosphorylation sites to disrupt mTOR regulation reveal the diverse roles played by the AMPK–mTOR axis. These regulatory events complement the direct regulation of metabolic enzymes by AMPK to efficiently rewire the cellular metabolism during energetic stress. However, the multifaceted role of AMPK in cellular homeostasis extends beyond its traditional metabolic functions by also impacting various non-metabolic processes, highlighting its broad influence on overall cellular physiology and health.

## Knock-in mouse models to explore non-metabolic roles of AMPK

### CRY1 in AMPK-mediated circadian rhythm

Mammalian circadian clocks synchronize behavior and physiology with daily light-dark cycles by orchestrating the rhythmic transcription of genes (Stephan and Zucker, 1972). The central clock in the brain is entrained by light, whereas clocks in peripheral tissues, such as those in the liver, are influenced by daily feeding patterns (Damiola et al., 2000; Schibler et al., 2003; Stokkan et al., 2001). AMPK is a nutrient-responsive signaling molecule that communicates metabolic signals to circadian clocks by initiating the phosphorylation of serine 71 in clock component cryptochrome-1 (CRY1), causing its subsequent degradation (Lamia et al., 2009). The control of the stability of CRY1 through AMPK phosphorylation is important for the circadian modulation of various metabolic processes, including body weight, glucose homeostasis, running endurance and feeding behavior (Andersson et al., 2004; Foretz et al., 2005; Minokoshi et al., 2004; Narkar et al., 2008; Villena et al., 2004; Viollet et al., 2003). AMPK activation in mice reduces endogenous CRY1 protein levels in liver nuclei, leading to decreased amplitude of circadian transcripts and to disrupted circadian rhythms (Lamia et al., 2009). In AMPK knockout mice and mouse embryonic fibroblasts, loss of AMPK signaling results in the disturbance of circadian rhythms and clock gene expression and in the stabilization of cryptochromes, indicating that AMPK plays a robust role in the metabolic regulation of light-independent peripheral circadian clocks, consistent with the role of feeding anticipation in regulating circadian rhythms (Lamia et al., 2009; Lee and Kim, 2013; Um et al., 2011; Vieira et al., 2008).

To investigate the importance of CRY1 regulation by AMPK, Vaughan et al. (2019) generated mice that carry alanine mutations in serine 71 of CRY1 (*Cry1^S71A^*). Surprisingly, under normal physiological conditions, the phosphorylation of CRY1 serine 71 was dispensable for the regulation of both central and peripheral circadian rhythms. There were no significant changes observed in circadian rhythm-related behavior nor in the molecular circadian rhythms in *Cry1^S71A^* mice. However, female *Cry1^S71A^* mice exhibited decreased voluntary locomotor activity compared to wild-type mice, suggesting a potential role in regulating voluntary activity levels (Vaughan et al., 2019). Thus, CRY1 phosphorylation by AMPK might be important in the response to metabolic challenges or to other aspects of physiology related to voluntary activity levels. The control of transcriptional networks by AMPK extends beyond the transcription factor CRY1 to also include chromatin remodeling via the histone acetyltransferase P300 (also known as EP300) to globally influence transcription.

### P300 in AMPK-mediated transcription

P300 is a multifunctional coactivator that activates transcription via chromatin remodeling and by binding to transcription factors that regulate cell growth, differentiation and metabolism, including peroxisome proliferator-activated receptor α (PPARα or PPARA), HNF1α (HNF1A) and RORA (Lau et al., 1999; Ogryzko et al., 1996; Shikama et al., 1997; Soutoglou et al., 2000; Zhang et al., 2016). Serine 89 of P300 is a target of multiple kinase cascades, including protein kinase C (PKC), SIK2 and AMPK pathways. The phosphorylation of serine 89 modulates the interaction of P300 with its coactivators to dictate P300 function (Liu et al., 2008; Yang et al., 2001; Yuan and Gambee, 2000; Yuan et al., 2002; Bricambert et al., 2010). For example, the interaction of P300 with β-catenin (CTNNB1) regulates the expression of differentiation-associated genes and is controlled by phosphorylation at serine 89 (Miyabayashi et al., 2007; Rieger et al., 2016). In the case of AMPK, this phosphorylation event on P300 modulates the transcriptional activity of nuclear hormone receptors (Yang et al., 2001).

In an effort to understand the role that P300 serine 89 plays *in vivo*, Lai et al. (2021) generated knock-in mice that carry a single amino acid mutation in P300, which converts serine 89 to alanine (*P300^S89A^*). This mutation abrogated the phosphorylation-dependent increase in the association of β-catenin with P300, reducing Wnt signaling and increasing the sensitivity of the mutant mice to intestinal injury with dextran sodium sulfate treatment and colorectal cancer. A decreased association of P300 with 14-3-3 proteins was also observed, which could have various consequences, including changes in protein conformation, stability and cellular localization (Darling et al., 2005; Obsilova and Obsil, 2022). Transcriptional and proteomic analysis further revealed the effects of the *P300^S89A^* mutation on mitochondrial dysfunction and oxidative phosphorylation, as well as on differentiation and the endocytic pathway. Of note, the bile acid transporter protein FABP6 was also downregulated in *P300^S89A^* knock-in mice, and FABP6 downregulation was required for the efficient absorption and transport of bile acids in the distal intestine (Lai et al., 2021). FABP6 is directly regulated by PPARα, which is coactivated by P300 and can be phosphorylated and activated by AMPK to regulate lipid metabolism (Estadual De Maringá et al., 2020; Wolfrum et al., 2001; Zhang et al., 2016). Thus, further studies using *P300^S89A^* knock-in mice could provide new insights into the transcriptional regulation of metabolism and into the role of the AMPK–P300 axis in development and disease. Besides the roles of AMPK in transcriptional regulation, it can also impact other physiological processes such as vasoconstriction (Box 2).

### ACE2 in AMPK-mediated pulmonary hypertension resistance

The renin–angiotensin system (RAS, Box 2) is a vitally important regulator of arterial blood pressure and has been implicated in obesity and hyperlipidemia (Richey et al., 1999; Szczepanska-Sadowska et al., 2018; Yvan-Charvet et al., 2005). Within the RAS, two opposing axes regulate angiotensin (Box 2) biology and function. In the classical arm, the ACE1 (or ACE)–angiotensin II (Ang II, encoded by *AGT*)–angiotensin II type-1 receptor (AGTR1 or AT1) axis functions to produce angiotensin and stimulate vasoconstriction. In the protective arm, angiotensin-converting enzyme 2 (ACE2) facilitates the conversion of Ang II into angiotensin 1-7 (Ang-1-7). Ang-1-7 subsequently interacts with Mas (or MAS1), a G protein-coupled receptor, to provide anti-vasoconstriction, anti-inflammation and anti-fibrotic effects (Frantz et al., 2018; Jiang et al., 2014; Santos et al., 2003, 2008; Tipnis et al., 2000). Thus, an increase in ACE2 function is associated with the amelioration of pulmonary hypertension (Ferreira et al., 2009; Johnson et al., 2012).

AMPK has been investigated as a potential regulator of the RAS, due to the association of the RAS with obesity, and AMPK has been found to phosphorylate ACE2 on serine 680 (Liu et al., 2019; Zhang et al., 2018). The AMPK-mediated phosphorylation of ACE2 improves ACE2 stability by inhibiting its ubiquitination and subsequent degradation by murine double minute 2 (MDM2). This increased ACE2 stability leads to increased Ang-1-7 levels and to increased bioavailability of nitric oxide, which helps protect against pulmonary hypertension (Shen et al., 2020; Zhang et al., 2018). Zhang et al. (2018) generated a gain-of-function mouse in which serine 680 of ACE2 was converted to aspartic acid, which is phosphomimetic and increases ACE2 protein levels (*Ace2^S680D^*). These mice were resistant to pulmonary hypertension, whereas *Ace2* knockout mice showed exacerbated pulmonary hypertension, highlighting a protective role for the AMPK-dependent phosphorylation of ACE2. Corroboratively, the levels of ACE2, AMPK phosphorylation at T172 and ACE2 phosphorylation at S680 were reduced in human lungs afflicted by idiopathic pulmonary arterial hypertension, indicating impaired ACE2 phosphorylation and further supporting the importance of the AMPK–ACE2 axis in pulmonary hypertension pathogenesis (Zhang et al., 2018). Zhang et al. (2018) also generated an ACE2 mouse model that mimics serine 680 dephosphorylation, in which serine 680 is converted to leucine (*Ace2^S680L^*). *Ace2^S680L^* mice displayed increased susceptibility to pulmonary hypertension (in contrast to *Ace2^S680D^* mice), reduced ACE2 stability and lower ACE2 levels. These results indicate that crosstalk occurs between AMPK and MDM2 in the pathogenesis of pulmonary hypertension (Shen et al., 2020). AMPK phosphorylation and regulation of ACE2 therefore presents a potential therapeutic target for pulmonary hypertension. The role of AMPK in the circulatory system is not restricted to just pulmonary hypertension and vasoconstriction, but it is also implicated in the innate immune response triggered by TANK-binding kinase 1 (TBK1).

### TBK1 in AMPK-mediated innate immunity

TBK1, a member of the IκB kinase family, is a serine/threonine protein kinase that is involved in the regulation of various cellular processes, including the immune response, inflammation, autophagy and cell survival (Herhaus, 2021; Zhou et al., 2020). TBK1 plays a vital role in host defense against pathogens and in maintaining cellular integrity and function. It is primarily known for its role in the induction of type I interferon production in response to viral infection and other immune stimuli (Revach et al., 2020; Zhou et al., 2020). It phosphorylates transcription factors, such as interferon regulatory factors 3 (IRF3) and 7 (IRF7), leading to their activation and to the subsequent expression of type I interferons and other antiviral genes (Herhaus, 2021; Ma et al., 2012; Zhou et al., 2020). A vital component of the innate immune system is the activation of nucleic acid sensing in cells, including by the stimulator of interferon genes (STING or STING1)–cyclic GMP-AMP synthase (c-GAS) pathway (Box 2) (Chen et al., 2016b; Roers et al., 2016). Nucleic acid sensing can trigger various biological processes and responses, including autophagy and mitochondrial dynamics (Maelfait et al., 2020), which lie downstream of AMPK. TBK1 can be phosphorylated and activated by AMPK at serine 511 to drive IRF3 recruitment and assembly of mitochondrial antiviral signaling protein (MAVS; Box 2) or STING signalosomes to initiate innate immune response (Zhang et al., 2022).

Zhang et al. (2022) explored the direct phosphorylation of TBK1 by AMPK to provide insights into the molecular basis of the AMPK–TBK1 axis with relevance to antiviral defense. They generated a knock-in mouse that carries a serine 511 to glutamic acid phosphomimetic mutation in TBK1 (*Tbk1^S511E^*). They also generated a mouse with serine 511 to alanine mutation in TBK1 (*Tbk1^S511A^*), which cannot be phosphorylated by AMPK and disrupts the AMPK–TBK1 axis. Both mouse lines were studied to investigate the *in vivo* functions of the AMPK–TBK1 pathway. Homozygotes of both genotypes appeared normal yet exhibited distinct responses to viral RNA sensing- and nucleic acid-induced immune activation. Whereas *Tbk1^S511E^* mice showed enhanced antiviral responses and reduced viral replication, *Tbk1^S511A^* mice displayed compromised immune sensing and increased viral susceptibility. Notably, herpes simplex virus 1 (HSV-1) viral loads were decreased in the eyelids of *Tbk1^S511E^* mice, and these mice showed improved ocular disease phenotypes as they had no eye swelling or eye closure compared to that in wild-type mice in response to HSV-1 corneal infection (Zhang et al., 2022). These findings underscore the critical role of the AMPK–TBK1 axis in the innate immune response to viral infections. Thus, in addition to the regulation of metabolic pathways, AMPK can also regulate non-metabolic pathways to support organismal health.

## Conclusions

Phosphorylation events by AMPK are critical for its function in a spectrum of both physiological and pathological contexts. The investigation of mice bearing point mutations at these crucial phosphorylation sites has provided mechanistic insight into several diseases. With the advent of phosphorylation-mutant mice, we can now state that phosphorylation of ACC1 and ACC2 is required for inhibition of *de novo* lipogenesis and activation of FAO to prevent fatty liver, diabetes and hepatocellular carcinoma. Similarly, mouse models now substantiate that phosphorylation of TBC1D1 is a critical regulator of glucose uptake by the muscle, whereas HMGCR phosphorylation controls cholesterol levels, both of which contribute to the prevention of fatty liver disease and obesity. The ability of AMPK activators to ameliorate these metabolic syndromes has also been mechanistically dissected to reveal the necessity for RAPTOR and HMGCR phosphorylation in the therapeutic effects of these drugs. Thus, these mouse models can aid in the clarification of how AMPK impacts metabolic diseases and of the underlying molecular mechanisms by which AMPK exerts its effects.

Some AMPK mutant mice also offer us invaluable insights into the fundamental mechanisms that govern normal physiological processes. For instance, mice with mutations in RAPTOR and WDR24 (in the GATOR2 complex) allow researchers to investigate the consequences of impaired AMPK signaling for the coordination and maintenance of metabolic homeostasis through mTORC1 signaling. Other phosphorylation-mutant mice have highlighted the role of AMPK in voluntary locomotor activity and the beneficial effects of exercise. Although AMPK is known to be upregulated during exercise, studies of FNIP1, TBC1D1 and TBC1D4 knock-in mouse models have revealed the importance of AMPK for mitochondrial homeostasis and for the regulation of insulin and glucose levels during exercise, thereby adjusting fuel utilization and supporting exercise endurance.

Future studies of these mice will help us to more fully delineate the mechanisms of AMPK-dependent processes. So far, these mice have allowed us to identify the importance of these processes *in vivo* and to manipulate their associated signaling pathways in various contexts. However, these mice have also revealed some of the hurdles involved in using phosphorylation-mutant mice to unravel the roles of AMPK. AMPK is currently known to phosphorylate a wide variety of targets involved in all facets of cellular biology. Although many of these sites have been validated, large proteome-wide screens indicate that AMPK has an even larger number of substrates that remain to be validated and explored. However, the ease of developing phosphorylation-mutant mice has led to instances where mice are generated without thorough investigation or validation of the sites. Specifically, many AMPK phosphorylation sites, including those in TBC1D4, ACE2 and WDR24, have not undergone testing to assess the impact of mutations on interactions, structure or activity, nor has independent validation of some of these sites been conducted. Consequently, the generation of phosphorylation-mutant mice does not necessarily indicate the priority or importance of the target in the realm of AMPK biology. Thus, determining which AMPK targets are the most crucial to assess in a mouse model remains a challenging issue.

The number of mouse models required to fully investigate AMPK biology increases even more when there is a need to explore models with both gain and loss of phosphorylation. Studies of models with gain and loss of phosphorylation for ACE2, TBK1, WDR24 and FNIP1 highlight how both types of mutations can reveal overlapping and divergent functions and roles. For example, *Ace2^S680D^* (phosphomimetic state) and *Ace2^S680L^* (phosphorylation-deficient state) mice both implicate AMPK in pulmonary hypertension, whereas *Tbk1^S511A^* (phosphorylation-deficient state) and *Tbk1^S511E^* (phosphomimetic state) mice show opposing effects on viral immunity. Hence, the multitude of phosphorylation-mutant mouse models required to comprehensively understand AMPK biology is vast, emphasizing the need to discern and select optimal targets.

Additionally, phosphorylation events on a single serine might be induced or influenced by other kinases apart from AMPK. As observed in the P300 mouse model, serine 89 is a known target of multiple kinase cascades in addition to AMPK, including PKC and SIK2 (Yuan and Gambee, 2000; Yuan et al., 2002; Bricambert et al., 2010). Although the authors investigated how this phosphorylation event altered β-catenin signaling, some of the phenotypes could also be ascribed to AMPK phosphorylation, including mitochondrial dysfunction and oxidative phosphorylation. In addition, many AMPK phosphorylation sites are contained within the consensus sites for other kinases. For example, AMPK and PKA phosphorylate RAPTOR at serine 792 and serine 791, respectively, with overlapping consensus sites (Le et al., 2023). Similarly, TBK1 can be phosphorylated at serine 511 and serine 510 by AMPK and AKT, respectively, with distinct and opposite effects (Wu et al., 2019; Zhang et al., 2022). However, the impact of serine mutations at these sites has not been explored to understand the impact on phosphorylation at the alternate sites. Thus, it is possible that mutating serine to alanine, aspartic acid or glutamic acid has indirect effects on these other signaling pathways. For instance, mutation of RAPTOR serine 792 to alanine could also impair PKA signaling, resulting in changes in growth and proliferation not attributable to AMPK.

To address the issue of potential AMPK-independent effects, investigators need to corroborate findings using multiple comparisons and techniques. First, the phenotypes of knock-in mice to AMPK knockout or knock-in controls should be compared, as can be done when comparing the high glycogen levels in the activating *Ampkg3^R225Q^* knock-in mouse model with the lower glycogen levels in the phospho-mutant *Mck-Fnip1^S220A^* mice (Barnes et al., 2004). Second, the phenotypes of knock-in mice in the context of AMPK activators should be evaluated, as was done in the case of metformin treatment of *Pfkfb2^S468A;S485A^* mice, which revealed an AMPK-independent role in the anti-fibrotic phenotype (Harley et al., 2023). Finally, validation that phosphorylation of alternate sites is not altered should be performed by using either western blotting or mass spectrometry (Box 2). It is also necessary to ensure that other functions and interactions of the mutated protein are intact to ensure that structural integrity is maintained, e.g. RAPTOR binding to mTOR is maintained in the presence of *Raptor^S722A;S792A^* mutations (Gwinn et al., 2008). Overall, these common phosphorylation and consensus sites make it essential to consider the context in which phenotypes are evaluated to determine their dependency on AMPK.

Due to the complex interplay between signaling pathways, it might also be necessary to explore combinatorial knock-in mouse models to uncover robust phenotypes, as suggested by the modest effects of the *Raptor^S722A;S792A^* knock-in mouse model in the presence of wild-type TSC2 (Van Nostrand et al., 2020). Although the authors used *Tcs2* knockout cells to reveal the relative importance of RAPTOR phosphorylation, a complete understanding of how the regulation of mTORC1 by AMPK impacts physiology will require the development of TSC2 phosphorylation-mutant mouse models to mitigate the high basal levels of mTORC1 caused by TSC2 loss. The subsequent combination of RAPTOR and TSC2 phosphorylation-mutant models with the *Wdr24^S155A^* mutant mouse model would help to more fully dissect AMPK and mTORC1 signaling. This capability and, indeed, necessity to combine various phosphorylation-mutant mouse models extends beyond AMPK and mTORC1 signaling and increases the complexity of studying AMPK using these mouse models.

In addition to the challenges of creating and analyzing novel phosphorylation-mutant mouse models, the species-specific functions of AMPK modulation pose a hurdle when extrapolating phenotypes from mouse models to human pathology. Factors such as variations in isoforms, drug responses, feeding and/or fasting patterns, and metabolic rates can all influence how changes in AMPK signaling affect pathological outcomes. Nonetheless, many critical signaling events between species are conserved, evidenced by the preservation of AMPK phosphorylation sites. Thus, although direct correlations may not always be established, observations of how signaling alterations impact phenotype offer valuable insights into fundamental biological processes in humans. For instance, ACC phosphorylation influencing fatty liver disease and liver cancer development provided the preclinical evidence to support clinical trials of ACC phosphorylation inhibitors in patients with liver disease (Batchuluun et al., 2022).

Alternatively, although mouse models are commonly used for studying complex biological processes, other genetically tractable organisms, such as *Drosophila melanogaster* (fruit flies), *Caenorhabditis elegans* (roundworms) and zebrafish, offer unique advantages in AMPK research in metabolism, cell polarity, autophagy and oxidative stress. For instance, studies have shown that genetic manipulation of AMPK activity in fruit flies and worms affects nutrient storage, FAO, lifespan and stress resistance (Beale, 2008; Jacobs et al., 2020; Sinnett and Brenman, 2016; Stancu, 2015). Moreover, zebrafish models of metabolic disorders, such as obesity and diabetes, have been used to investigate the therapeutic potential of AMPK activators to improve glucose homeostasis and lipid metabolism (Ghaddar and Diotel, 2022; Gut et al., 2017; Wiggenhauser and Kroll, 2018). By leveraging the experimental advantages offered by these genetically modified models and combining them with studies using mutant mouse models, researchers can uncover intriguing possibilities for addressing physiological conditions with AMPK modulators.

Overall, AMPK phosphorylation-mutant mouse models have shed new light on the ability of AMPK to modulate numerous pathological contexts. These investigations have not only deepened our understanding of the role of AMPK in metabolic regulation, but they have also shed light on its involvement in broader physiological functions, including cell growth, autophagy and innate immunity (Box 2). In addition to understanding basic AMPK biology, these mouse models can serve as preclinical tools for testing and developing drugs for metabolic diseases that can selectively target AMPK in specific tissues or cellular contexts to minimize potential side effects. Moreover, understanding the impact of these specific phosphorylation mutations on metabolic regulation can inform precision medicine approaches. For example, understanding the circumstances in which the phosphorylation of ACCs by AMPK is essential can identify when the ACC inhibitor ND646, which targets the AMPK phosphorylation site, would be the most beneficial (Lally et al., 2019; Svensson et al., 2016). The use of advanced genetic and molecular techniques, coupled with the generation of more sophisticated mouse models, will undoubtedly facilitate the exploration of AMPK in disease to create tailored therapeutic strategies and interventions for combating the rising tide of metabolic disorders to improve human health.
